# 1,1-Dibenzyl-3-(3-chloro­benzo­yl)thio­urea

**DOI:** 10.1107/S1600536811023191

**Published:** 2011-06-18

**Authors:** Mohd Faizal Md Nasir, Ibrahim N. Hassan, Bohari Yamin, W. R. W Daud, Mohammad B. Kassim

**Affiliations:** a Fuel Cell Institute, Universiti Kebangsaan Malaysia, 43600 Selangor, Malaysia; b School of Chemical Sciences & Food Technology, Faculty of Science & Technology, Universiti Kebangsaan Malaysia, 43600 Selangor, Malaysia; c Department of Chemical and Process Engineering, Faculty of Engineering and Built Environment, Universiti Kebangsaan Malaysia, 43600 Selangor, Malaysia

## Abstract

In the title compound, C_22_H_19_ClN_2_OS, the thiono and carbonyl groups are *trans* positioned with respect to a partially double C—N bond. The amide group is twisted relative to the thio­urea fragment, forming a dihedral angle of 46.75 (11)°. In the crystal, inter­molecular N—H⋯S and C—H⋯O hydrogen bonds link the mol­ecules into a one-dimensional polymeric structure parallel to the *c* axis.

## Related literature

For related structures and background references, see: Al-abbasi & Kassim (2011 ▶); Nasir *et al.* (2011 ▶). For metal complexes of benzoyl­thio­ureas, see: Weiqun *et al.* (2005 ▶); Circu *et al.* (2009 ▶). For the synthetic procedure, see: Hassan *et al.* (2008 ▶).
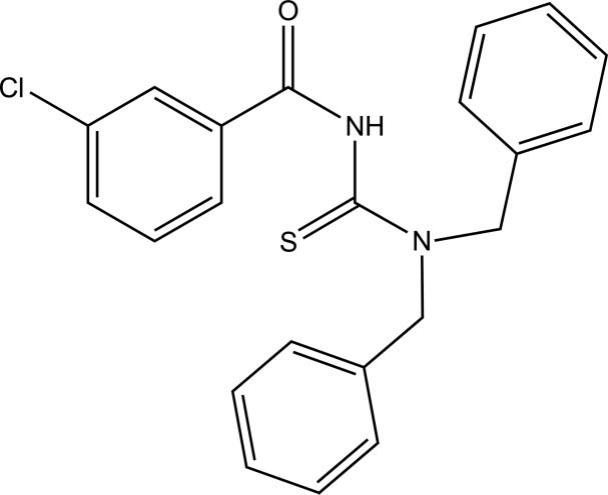


## Experimental

### 

#### Crystal data

C_22_H_19_ClN_2_OS
*M* *_r_* = 394.90
Triclinic, 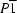

*a* = 9.503 (4) Å
*b* = 9.650 (4) Å
*c* = 12.487 (5) Å
α = 72.422 (8)°
β = 72.869 (9)°
γ = 69.463 (8)°
*V* = 999.1 (7) Å^3^
*Z* = 2
Mo *K*α radiation
μ = 0.31 mm^−1^
*T* = 298 K
0.28 × 0.17 × 0.13 mm

#### Data collection

Bruker SMART APEX CCD area-detector diffractometer
Absorption correction: multi-scan (*SADABS*; Bruker, 2000 ▶) *T*_min_ = 0.918, *T*_max_ = 0.961
13666 measured reflections
5000 independent reflections
2879 reflections with *I* > 2σ(*I*)
*R*_int_ = 0.036

#### Refinement

*R*[*F* ^2^ > 2σ(*F* ^2^)] = 0.054
*wR*(*F* ^2^) = 0.147
*S* = 1.03
5000 reflections
244 parameters
H-atom parameters constrained
Δρ_max_ = 0.34 e Å^−3^
Δρ_min_ = −0.31 e Å^−3^

### 

Data collection: *SMART* (Bruker, 2000 ▶); cell refinement: *SAINT* (Bruker, 2000 ▶); data reduction: *SAINT*; program(s) used to solve structure: *SHELXS97* (Sheldrick, 2008 ▶); program(s) used to refine structure: *SHELXL97* (Sheldrick, 2008 ▶); molecular graphics: *SHELXTL* (Sheldrick, 2008 ▶); software used to prepare material for publication: *SHELXTL*, *PARST* (Nardelli, 1995 ▶) and *PLATON* (Spek, 2009 ▶).

## Supplementary Material

Crystal structure: contains datablock(s) I, global. DOI: 10.1107/S1600536811023191/gk2383sup1.cif

Structure factors: contains datablock(s) I. DOI: 10.1107/S1600536811023191/gk2383Isup2.hkl

Supplementary material file. DOI: 10.1107/S1600536811023191/gk2383Isup3.cml

Additional supplementary materials:  crystallographic information; 3D view; checkCIF report

## Figures and Tables

**Table 1:** Hydrogen-bond geometry (Å, °)

*D*—H⋯*A*	*D*—H	H⋯*A*	*D*⋯*A*	*D*—H⋯*A*
N1—H1⋯S1^i^	0.86	2.74	3.410 (2)	136
C15—H15⋯O1^ii^	0.93	2.50	3.421 (3)	170

Symmetry codes: (i) 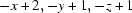; (ii) 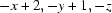.

**Fig. 1.**
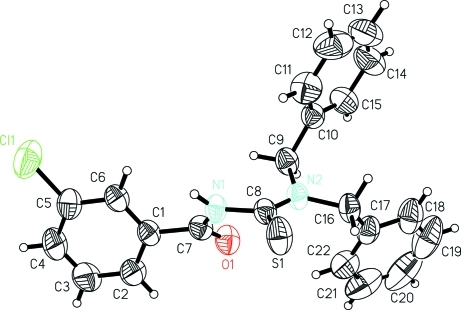
The molecular structure of the title compound with displacement ellipsoids drawn at the 50% probability level.

**Fig. 2.**
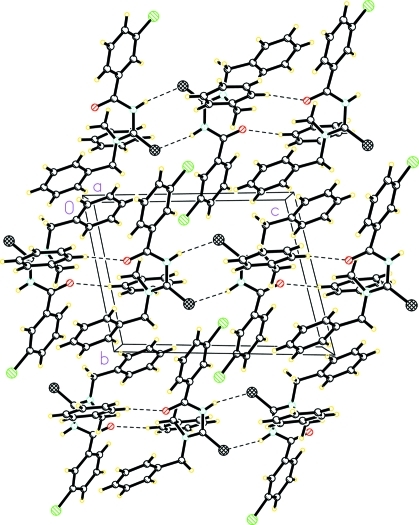
A packing diagram of the title compound down the a-axis showing the intermolecular hydrogen bonds N1—H1···S1 (-x + 2, -y + 1, -z + 1) and C15—H15··· O1 (-x + 2, -y + 1, -z).

## supplementary crystallographic information

### Comment

Benzoylthiourea compounds contain strong donor groups (carbonyl and thioamide) which make them very attractive ligands in coordination chemistry. These ligands react with transition metals, mostly in monoanionic and bidentate form by deprotonation of the amide group, forming neutral complexes with S, O-coordination (Weiqun *et al.*, 2005; Circu *et al.*, 2009).

The title compound, I, is a thiourea derivative analogous to our previously reported compounds (Nasir *et al.*, 2011; Al-abbasi & Kassim, 2011). The thiono S and the carbonyl O atoms are *trans* positioned at a partially double N1-C8 bond with C7N1C8S1 torsion angle of 127.37 (18)°. The dihedral angle between the mean planes of the thiourea (S1/N1/N2/C8) and the amide group (O1/N1/C1/C7) is 46.75 (11)°. The mean planes of the dibenzylamine (C9/C10/C11/C12/C13/C14/C15 and C16/C17/C18/C19/C19/C20/C21) make an angle of 20.54 (13)°.

Intermolecular N—H···S and C—H···O hydrogen bonds link the molecules into a one dimensional polymeric structure parallel to the *c*-axis.

### Experimental

The title compound was prepared according to a previously reported compound (Hassan *et al.*, 2008). A colourless crystal, suitable for X-ray crystallography, was obtained by a slow evaporation from a mixture of acetone/ethanol solution at room temperature (yield 80%).

### Refinement

All H atoms were postioned geometrically with C-H bond lengths in the range 0.93 - 0.97 Å and N-H bond of 0.86 Å,.and refined in the riding model approximation with *U*_iso_(H)=1.2*U*_eq_(C,N), except for methyl group where *U*_iso_(H)= 1.5*U*_eq_(C).

### Crystal data

**Table:**

C_22_H_19_ClN_2_OS	*Z* = 2
*M**_r_* = 394.90	*F*(000) = 412
Triclinic, *P*1	*D*_x_ = 1.313 Mg m^−^^3^
Hall symbol: -P 1	Melting point: 409.15 K
*a* = 9.503 (4) Å	Mo *K*α radiation, λ = 0.71073 Å
*b* = 9.650 (4) Å	Cell parameters from 1114 reflections
*c* = 12.487 (5) Å	θ = 2.3–28.5°
α = 72.422 (8)°	µ = 0.31 mm^−^^1^
β = 72.869 (9)°	*T* = 298 K
γ = 69.463 (8)°	Block, colourless
*V* = 999.1 (7) Å^3^	0.28 × 0.17 × 0.13 mm

### Data collection

**Table:**

Bruker SMART APEX CCD area-detector diffractometer	5000 independent reflections
Radiation source: fine-focus sealed tube	2879 reflections with *I* > 2σ(*I*)
graphite	*R*_int_ = 0.036
ω scans	θ_max_ = 28.5°, θ_min_ = 2.3°
Absorption correction: multi-scan (*SADABS*; Bruker, 2000)	*h* = −12→12
*T*_min_ = 0.918, *T*_max_ = 0.961	*k* = −12→12
13666 measured reflections	*l* = −16→16

### Refinement

**Table:**

Refinement on *F*^2^	Primary atom site location: structure-invariant direct methods
Least-squares matrix: full	Secondary atom site location: difference Fourier map
*R*[*F*^2^ > 2σ(*F*^2^)] = 0.054	Hydrogen site location: inferred from neighbouring sites
*wR*(*F*^2^) = 0.147	H-atom parameters constrained
*S* = 1.03	*w* = 1/[σ^2^(*F*_o_^2^) + (0.0679*P*)^2^ + 0.0522*P*] where *P* = (*F*_o_^2^ + 2*F*_c_^2^)/3
5000 reflections	(Δ/σ)_max_ = 0.001
244 parameters	Δρ_max_ = 0.34 e Å^−^^3^
0 restraints	Δρ_min_ = −0.31 e Å^−^^3^

### Special details

**Table:**

Geometry. All e.s.d.'s (except the e.s.d. in the dihedral angle between two l.s. planes) are estimated using the full covariance matrix. The cell e.s.d.'s are taken into account individually in the estimation of e.s.d.'s in distances, angles and torsion angles; correlations between e.s.d.'s in cell parameters are only used when they are defined by crystal symmetry. An approximate (isotropic) treatment of cell e.s.d.'s is used for estimating e.s.d.'s involving l.s. planes.
Refinement. Refinement of *F*^2^ against ALL reflections. The weighted *R*-factor *wR* and goodness of fit *S* are based on *F*^2^, conventional *R*-factors *R* are based on *F*, with *F* set to zero for negative *F*^2^. The threshold expression of *F*^2^ > σ(*F*^2^) is used only for calculating *R*-factors(gt) *etc*. and is not relevant to the choice of reflections for refinement. *R*-factors based on *F*^2^ are statistically about twice as large as those based on *F*, and *R*- factors based on ALL data will be even larger.

### Fractional atomic coordinates and isotropic or equivalent isotropic displacement parameters (Å^2^)

**Table:**

	*x*	*y*	*z*	*U*_iso_*/*U*_eq_	
S1	0.89445 (7)	0.70137 (6)	0.39746 (5)	0.0651 (2)	
Cl1	1.24059 (9)	−0.15952 (8)	0.53858 (7)	0.0984 (3)	
O1	1.15882 (17)	0.42880 (17)	0.15716 (13)	0.0614 (4)	
N1	1.01426 (17)	0.43643 (17)	0.33839 (14)	0.0475 (4)	
H1	0.9940	0.3826	0.4067	0.057*	
N2	0.86120 (18)	0.62690 (17)	0.21995 (14)	0.0474 (4)	
C8	0.9214 (2)	0.5863 (2)	0.31191 (17)	0.0454 (5)	
C1	1.2465 (2)	0.2283 (2)	0.30830 (18)	0.0476 (5)	
C7	1.1378 (2)	0.3712 (2)	0.25868 (19)	0.0482 (5)	
C6	1.1962 (2)	0.1167 (2)	0.39517 (18)	0.0517 (5)	
H6	1.0920	0.1315	0.4278	0.062*	
C10	0.6590 (2)	0.5487 (2)	0.19232 (18)	0.0502 (5)	
C9	0.8302 (2)	0.5205 (2)	0.1730 (2)	0.0541 (5)	
H9A	0.8767	0.4170	0.2104	0.065*	
H9B	0.8751	0.5338	0.0914	0.065*	
C16	0.7941 (2)	0.7885 (2)	0.17143 (19)	0.0542 (5)	
H16A	0.8138	0.8494	0.2115	0.065*	
H16B	0.6835	0.8095	0.1842	0.065*	
C17	0.8578 (3)	0.8343 (2)	0.04457 (19)	0.0569 (6)	
C5	1.3029 (3)	−0.0172 (2)	0.43277 (19)	0.0607 (6)	
C15	0.5883 (3)	0.5901 (3)	0.1013 (2)	0.0658 (6)	
H15	0.6472	0.5954	0.0269	0.079*	
C4	1.4577 (3)	−0.0385 (3)	0.3883 (2)	0.0708 (7)	
H4	1.5287	−0.1283	0.4150	0.085*	
C2	1.4025 (2)	0.2061 (2)	0.2627 (2)	0.0633 (6)	
H2	1.4370	0.2804	0.2039	0.076*	
C3	1.5060 (3)	0.0740 (3)	0.3045 (2)	0.0751 (8)	
H3	1.6107	0.0609	0.2752	0.090*	
C22	1.0129 (3)	0.7936 (3)	−0.0005 (2)	0.0719 (7)	
H22	1.0805	0.7298	0.0466	0.086*	
C11	0.5686 (3)	0.5384 (3)	0.3020 (2)	0.0741 (7)	
H11	0.6143	0.5088	0.3649	0.089*	
C18	0.7596 (4)	0.9279 (3)	−0.0270 (2)	0.0797 (8)	
H18	0.6540	0.9551	0.0021	0.096*	
C14	0.4304 (3)	0.6240 (3)	0.1196 (3)	0.0860 (8)	
H14	0.3838	0.6519	0.0573	0.103*	
C21	1.0691 (4)	0.8466 (4)	−0.1150 (3)	0.0994 (11)	
H21	1.1744	0.8176	−0.1449	0.119*	
C19	0.8167 (6)	0.9815 (4)	−0.1415 (3)	0.1121 (13)	
H19	0.7500	1.0453	−0.1893	0.135*	
C20	0.9722 (7)	0.9406 (4)	−0.1848 (3)	0.1165 (15)	
H20	1.0111	0.9774	−0.2619	0.140*	
C12	0.4102 (4)	0.5717 (4)	0.3194 (3)	0.0922 (9)	
H12	0.3504	0.5633	0.3936	0.111*	
C13	0.3428 (3)	0.6166 (3)	0.2279 (4)	0.0907 (10)	
H13	0.2364	0.6424	0.2393	0.109*	

### Atomic displacement parameters (Å^2^)

**Table:**

	*U*^11^	*U*^22^	*U*^33^	*U*^12^	*U*^13^	*U*^23^
S1	0.0793 (4)	0.0510 (3)	0.0616 (4)	0.0073 (3)	−0.0289 (3)	−0.0249 (3)
Cl1	0.0963 (6)	0.0616 (4)	0.0962 (5)	−0.0036 (4)	−0.0078 (4)	0.0053 (4)
O1	0.0552 (9)	0.0614 (9)	0.0517 (9)	−0.0035 (7)	−0.0041 (7)	−0.0128 (7)
N1	0.0469 (9)	0.0392 (8)	0.0447 (9)	0.0005 (7)	−0.0070 (8)	−0.0106 (7)
N2	0.0481 (9)	0.0393 (9)	0.0529 (10)	−0.0012 (7)	−0.0168 (8)	−0.0152 (8)
C8	0.0391 (10)	0.0428 (11)	0.0480 (11)	−0.0037 (8)	−0.0078 (9)	−0.0122 (9)
C1	0.0418 (11)	0.0433 (11)	0.0541 (12)	−0.0008 (9)	−0.0097 (9)	−0.0197 (9)
C7	0.0428 (11)	0.0468 (11)	0.0532 (13)	−0.0074 (9)	−0.0068 (9)	−0.0181 (10)
C6	0.0427 (11)	0.0502 (12)	0.0567 (13)	−0.0010 (9)	−0.0083 (10)	−0.0210 (10)
C10	0.0497 (12)	0.0441 (11)	0.0553 (12)	−0.0084 (9)	−0.0111 (10)	−0.0149 (9)
C9	0.0539 (12)	0.0463 (11)	0.0630 (13)	−0.0027 (10)	−0.0180 (11)	−0.0217 (10)
C16	0.0556 (13)	0.0414 (11)	0.0614 (13)	0.0017 (9)	−0.0216 (11)	−0.0149 (10)
C17	0.0731 (15)	0.0413 (11)	0.0611 (14)	−0.0108 (11)	−0.0246 (12)	−0.0147 (10)
C5	0.0633 (15)	0.0476 (12)	0.0619 (14)	−0.0004 (10)	−0.0125 (11)	−0.0180 (11)
C15	0.0576 (14)	0.0784 (17)	0.0654 (15)	−0.0165 (12)	−0.0158 (12)	−0.0224 (13)
C4	0.0574 (15)	0.0530 (14)	0.0874 (18)	0.0118 (12)	−0.0208 (14)	−0.0226 (13)
C2	0.0465 (12)	0.0501 (13)	0.0797 (16)	−0.0031 (10)	−0.0024 (11)	−0.0187 (12)
C3	0.0410 (13)	0.0644 (16)	0.106 (2)	0.0039 (11)	−0.0051 (13)	−0.0322 (16)
C22	0.0767 (18)	0.0659 (16)	0.0727 (17)	−0.0243 (14)	−0.0109 (14)	−0.0150 (13)
C11	0.0800 (18)	0.0793 (18)	0.0590 (15)	−0.0218 (15)	−0.0077 (13)	−0.0176 (13)
C18	0.110 (2)	0.0598 (15)	0.0720 (18)	−0.0091 (15)	−0.0449 (16)	−0.0109 (13)
C14	0.0605 (17)	0.101 (2)	0.108 (2)	−0.0165 (15)	−0.0323 (17)	−0.0332 (19)
C21	0.128 (3)	0.087 (2)	0.086 (2)	−0.058 (2)	0.019 (2)	−0.0300 (19)
C19	0.203 (4)	0.071 (2)	0.069 (2)	−0.033 (3)	−0.059 (3)	−0.0023 (17)
C20	0.221 (5)	0.078 (2)	0.060 (2)	−0.074 (3)	−0.007 (3)	−0.0133 (17)
C12	0.079 (2)	0.097 (2)	0.095 (2)	−0.0399 (18)	0.0271 (18)	−0.0401 (18)
C13	0.0533 (16)	0.085 (2)	0.144 (3)	−0.0193 (14)	−0.009 (2)	−0.051 (2)

### Geometric parameters (Å, °)

**Table:**

S1—C8	1.672 (2)	C5—C4	1.375 (3)
Cl1—C5	1.731 (3)	C15—C14	1.382 (4)
O1—C7	1.208 (3)	C15—H15	0.9300
N1—C7	1.392 (2)	C4—C3	1.365 (3)
N1—C8	1.402 (2)	C4—H4	0.9300
N1—H1	0.8600	C2—C3	1.373 (3)
N2—C8	1.326 (2)	C2—H2	0.9300
N2—C9	1.470 (3)	C3—H3	0.9300
N2—C16	1.471 (2)	C22—C21	1.375 (4)
C1—C6	1.384 (3)	C22—H22	0.9300
C1—C2	1.386 (3)	C11—C12	1.388 (4)
C1—C7	1.488 (3)	C11—H11	0.9300
C6—C5	1.383 (3)	C18—C19	1.377 (4)
C6—H6	0.9300	C18—H18	0.9300
C10—C15	1.374 (3)	C14—C13	1.359 (4)
C10—C11	1.382 (3)	C14—H14	0.9300
C10—C9	1.508 (3)	C21—C20	1.356 (5)
C9—H9A	0.9700	C21—H21	0.9300
C9—H9B	0.9700	C19—C20	1.371 (6)
C16—C17	1.507 (3)	C19—H19	0.9300
C16—H16A	0.9700	C20—H20	0.9300
C16—H16B	0.9700	C12—C13	1.357 (5)
C17—C22	1.372 (3)	C12—H12	0.9300
C17—C18	1.378 (3)	C13—H13	0.9300
			
C7—N1—C8	122.70 (17)	C10—C15—C14	120.6 (2)
C7—N1—H1	118.6	C10—C15—H15	119.7
C8—N1—H1	118.6	C14—C15—H15	119.7
C8—N2—C9	123.98 (17)	C3—C4—C5	119.2 (2)
C8—N2—C16	120.06 (17)	C3—C4—H4	120.4
C9—N2—C16	115.01 (16)	C5—C4—H4	120.4
N2—C8—N1	117.33 (17)	C3—C2—C1	119.8 (2)
N2—C8—S1	124.47 (15)	C3—C2—H2	120.1
N1—C8—S1	118.19 (14)	C1—C2—H2	120.1
C6—C1—C2	119.73 (19)	C4—C3—C2	121.1 (2)
C6—C1—C7	122.07 (18)	C4—C3—H3	119.5
C2—C1—C7	118.2 (2)	C2—C3—H3	119.5
O1—C7—N1	122.69 (19)	C17—C22—C21	120.4 (3)
O1—C7—C1	122.22 (18)	C17—C22—H22	119.8
N1—C7—C1	115.04 (18)	C21—C22—H22	119.8
C5—C6—C1	119.1 (2)	C10—C11—C12	120.7 (3)
C5—C6—H6	120.4	C10—C11—H11	119.7
C1—C6—H6	120.4	C12—C11—H11	119.7
C15—C10—C11	118.3 (2)	C19—C18—C17	120.4 (3)
C15—C10—C9	121.0 (2)	C19—C18—H18	119.8
C11—C10—C9	120.7 (2)	C17—C18—H18	119.8
N2—C9—C10	109.86 (16)	C13—C14—C15	120.4 (3)
N2—C9—H9A	109.7	C13—C14—H14	119.8
C10—C9—H9A	109.7	C15—C14—H14	119.8
N2—C9—H9B	109.7	C20—C21—C22	120.5 (3)
C10—C9—H9B	109.7	C20—C21—H21	119.7
H9A—C9—H9B	108.2	C22—C21—H21	119.7
N2—C16—C17	112.77 (16)	C20—C19—C18	119.9 (3)
N2—C16—H16A	109.0	C20—C19—H19	120.0
C17—C16—H16A	109.0	C18—C19—H19	120.0
N2—C16—H16B	109.0	C21—C20—C19	119.9 (3)
C17—C16—H16B	109.0	C21—C20—H20	120.1
H16A—C16—H16B	107.8	C19—C20—H20	120.1
C22—C17—C18	118.9 (2)	C13—C12—C11	119.9 (3)
C22—C17—C16	121.5 (2)	C13—C12—H12	120.1
C18—C17—C16	119.5 (2)	C11—C12—H12	120.1
C4—C5—C6	121.1 (2)	C12—C13—C14	120.2 (3)
C4—C5—Cl1	119.47 (18)	C12—C13—H13	119.9
C6—C5—Cl1	119.44 (18)	C14—C13—H13	119.9
			
C9—N2—C8—N1	26.1 (3)	C1—C6—C5—Cl1	−178.21 (16)
C16—N2—C8—N1	−165.58 (17)	C11—C10—C15—C14	1.5 (4)
C9—N2—C8—S1	−154.11 (16)	C9—C10—C15—C14	−176.4 (2)
C16—N2—C8—S1	14.2 (3)	C6—C5—C4—C3	−1.0 (4)
C7—N1—C8—N2	52.4 (3)	Cl1—C5—C4—C3	179.7 (2)
C7—N1—C8—S1	−127.36 (18)	C6—C1—C2—C3	−0.4 (3)
C8—N1—C7—O1	−12.5 (3)	C7—C1—C2—C3	−178.5 (2)
C8—N1—C7—C1	164.86 (17)	C5—C4—C3—C2	−1.2 (4)
C6—C1—C7—O1	−140.7 (2)	C1—C2—C3—C4	1.9 (4)
C2—C1—C7—O1	37.3 (3)	C18—C17—C22—C21	0.5 (4)
C6—C1—C7—N1	41.9 (3)	C16—C17—C22—C21	−174.9 (2)
C2—C1—C7—N1	−140.1 (2)	C15—C10—C11—C12	−1.1 (4)
C2—C1—C6—C5	−1.7 (3)	C9—C10—C11—C12	176.8 (2)
C7—C1—C6—C5	176.25 (19)	C22—C17—C18—C19	−1.0 (4)
C8—N2—C9—C10	110.5 (2)	C16—C17—C18—C19	174.5 (2)
C16—N2—C9—C10	−58.3 (2)	C10—C15—C14—C13	0.0 (4)
C15—C10—C9—N2	119.3 (2)	C17—C22—C21—C20	0.5 (4)
C11—C10—C9—N2	−58.6 (3)	C17—C18—C19—C20	0.5 (5)
C8—N2—C16—C17	127.7 (2)	C22—C21—C20—C19	−1.1 (5)
C9—N2—C16—C17	−63.0 (2)	C18—C19—C20—C21	0.6 (5)
N2—C16—C17—C22	−47.3 (3)	C10—C11—C12—C13	−0.7 (4)
N2—C16—C17—C18	137.3 (2)	C11—C12—C13—C14	2.3 (5)
C1—C6—C5—C4	2.5 (3)	C15—C14—C13—C12	−1.9 (5)

### Hydrogen-bond geometry (Å, °)

**Table:**

*D*—H···*A*	*D*—H	H···*A*	*D*···*A*	*D*—H···*A*
C16—H16A···S1	0.97	2.51	3.029 (3)	113
N1—H1···S1^i^	0.86	2.74	3.410 (2)	136
C15—H15···O1^ii^	0.93	2.50	3.421 (3)	170

Symmetry codes:  (i) −*x*+2, −*y*+1, −*z*+1; (ii) −*x*+2, −*y*+1, −*z*.

## References

[bb1] Al-abbasi, A. A. & Kassim, M. B. (2011). *Acta Cryst.* E**67**, o611. 10.1107/S1600536811004326 PMC3052143 21522368

[bb2] Bruker (2000). *SADABS*, *SMART* and *SAINT* Bruker AXS Inc., Madison, Wisconsin, USA.

[bb3] Circu, V., Ilie, M., Ilis, M., Dumitrascu, F., Neagoe, I. & Pasculescu, S. (2009). *Polyhedron*, **28**, 3739–3746.

[bb4] Hassan, I. N., Yamin, B. M. & Kassim, M. B. (2008). *Acta Cryst.* E**64**, o1727. 10.1107/S1600536808024896 PMC2960614 21201710

[bb5] Nardelli, M. (1995). *J. Appl. Cryst.* **28**, 659.

[bb6] Nasir, M. F. M., Hassan, I. N., Wan Daud, W. R., Yamin, B. M. & Kassim, M. B. (2011). *Acta Cryst.* E**67**, o1218. 10.1107/S1600536811014711 PMC3089282 21754516

[bb7] Sheldrick, G. M. (2008). *Acta Cryst.* A**64**, 112–122. 10.1107/S0108767307043930 18156677

[bb8] Spek, A. L. (2009). *Acta Cryst.* D**65**, 148–155. 10.1107/S090744490804362X PMC2631630 19171970

[bb9] Weiqun, Z., Wen, Y., Liqun, X. & Xianchen, C. (2005). *J. Inorg. Biochem.* **99**, 1314–1319. 10.1016/j.jinorgbio.2005.03.004 15917085

